# Cognitive assessment in myalgic encephalomyelitis/chronic fatigue syndrome (ME/CFS): a cognitive substudy of the multi-site clinical assessment of ME/CFS (MCAM)

**DOI:** 10.3389/fnins.2024.1460157

**Published:** 2024-11-01

**Authors:** Gudrun Lange, Jin-Mann S. Lin, Yang Chen, Elizabeth A. Fall, Daniel L. Peterson, Lucinda Bateman, Charles Lapp, Richard N. Podell, Benjamin H. Natelson, Andreas M. Kogelnik, Nancy G. Klimas, Elizabeth R. Unger

**Affiliations:** ^1^Pain and Fatigue Study Center, Department of Neurology, Icahn School of Medicine at Mount Sinai, New York, NY, United States; ^2^Division of High-Consequence Pathogens and Pathology, National Center for Emerging and Zoonotic Infectious Diseases, Centers for Disease Control and Prevention, Atlanta, GA, United States; ^3^Sierra Internal Medicine, Incline Village, NV, United States; ^4^Bateman Horne Center, Salt Lake City, UT, United States; ^5^Hunter-Hopkins Center, Charlotte, NC, United States; ^6^Richard N. Podell Medical, Summit, NJ, United States; ^7^Basis Diagnostics, Inc., Newark, CA, United States; ^8^Institute for Neuro Immune Medicine, Nova Southeastern University, Fort Lauderdale, FL, United States; ^9^VA Medical Center, Geriatric Research and Education Clinical Center, Miami, FL, United States

**Keywords:** myalgic encephalomyelitis/chronic fatigue syndrome (ME/CFS), computerized neurocognitive screening, longitudinal assessment, speed and accuracy of performance, executive function, physical exertion

## Abstract

**Introduction:**

Patients with Myalgic Encephalomyelitis/Chronic Fatigue Syndrome (ME/CFS) experience cognitive problems with attention, information processing speed, working memory, learning efficiency, and executive function. Commonly, patients report worsening of cognitive symptoms over time after physical and/or cognitive challenges. To determine, monitor, and manage longitudinal decrements in cognitive function after such exposures, it is important to be able to screen for cognitive dysfunction and changes over time in clinic and also remotely at home. The primary objectives of this paper were: (1) to determine whether a brief computerized cognitive screening battery will detect differences in cognitive function between ME/CFS and Healthy Controls (HC), (2) to monitor the impact of a full-day study visit on cognitive function over time, and (3) to evaluate the impact of exercise testing on cognitive dysfunction.

**Methods:**

This cognitive sub-study was conducted between 2013 and 2019 across seven U.S. ME/CFS clinics as part of the Multi-Site Clinical Assessment of ME/CFS (MCAM) study. The analysis included 426 participants (261 ME/CFS and 165 HC), who completed cognitive assessments including a computerized CogState Brief Screening Battery (CBSB) administered across five timepoints (T0-T4) at the start of and following a full day in-clinic visit that included exercise testing for a subset of participants (182 ME/CFS and 160 HC). Exercise testing consisted of ramped cycle ergometry to volitional exhaustion. The primary outcomes are performance accuracy and latency (performance speed) on the computerized CBSB administered online in clinic (T0 and T1) and at home (T2-T4).

**Results:**

No difference was found in performance accuracy between ME/CFS and HCs whereas information processing speed was significantly slower for ME/CFS at most timepoints with Cohen’s d effect sizes ranging from 0.3–0.5 (*p* < 0.01). The cognitive decline over time on all CBSB tasks was similar for patients with ME/CFS independent of whether exercise testing was included in the clinic visit.

**Conclusion:**

The challenges of a clinic visit (including cognitive testing) can lead to further cognitive deficits. A single short session of intense exercise does not further reduce speed of performance on any CBSB tasks.

## 1 Introduction

Myalgic encephalomyelitis/chronic fatigue syndrome (ME/CFS) is a complex, debilitating, multi-system illness with either sudden or gradual onset; its cause as yet unknown. Persons with ME/CFS have a substantial reduction or impairment in their ability to engage in pre-illness levels of activity that is accompanied by profound fatigue. The fatigue that they are experiencing is very different from just being tired. Other symptoms can include problems with sleep, thinking, concentrating, lightheadedness, dizziness, and pain (Institute of Medicine, 2015). ME/CFS affects about 836,000 to 3.3 million Americans and accounts for $18–51 billion of economic costs annually (Institute of Medicine, 2015; Vahratian et al., 2023; Bae and Lin, 2019).

Cognitive dysfunction in ME/CFS, a commonly reported symptom (Jason et al., 1999; Cvejic et al., 2016), has received significant attention over the past 40 years due to its disruptive effect on the professional and personal well-being of persons with ME/CFS (Christodoulou et al., 1998). The most commonly identified areas of cognitive difficulties include processing speed, attention, working memory, and learning efficiency (Cvejic et al., 2016; Sebaiti et al., 2022; Cockshell and Mathias, 2010). Over the years, neuropsychological studies have shown that cognitive dysfunction in ME/CFS is independent of mood disorders (Cockshell and Mathias, 2013; Majer et al., 2008; Robinson et al., 2019) and is not reflective of poor effort or motivation (Cvejic et al., 2016; Cockshell and Mathias, 2012; Lange et al., 2005).

Past studies have suggested that physical exertion (e.g., exercise) may further exacerbate cognitive impairments seen in ME/CFS (LaManca et al., 1998). However, findings are mixed due to small sample sizes and the use of different arrays of neuropsychological measures (LaManca et al., 1998; Marcel et al., 1996; Mahurin et al., 2004). In general, although variable, findings of these studies show that significant differences between persons with ME/CFS and healthy controls (HC) are observed mostly when latency/speed of completion is the outcome measure, not accuracy (Deluca et al., 2004; Lange et al., 2005).

There is a need for a large-scale case-control study to identify a brief computerized screening battery that is reliable, valid, and easy to administer to effectively determine presence, nature, and changes in cognitive function in ME/CFS over time and that can be administered in a traditional face-to-face setting and remotely at home. Remote, long-term online monitoring of cognitive function is an important clinical tool for the management of ME/CFS.

The Multi-site Clinical Assessment of ME/CFS (MCAM) study (Unger et al., 2017) provided this opportunity. This study enrolled a large sample of diverse and well-characterized patients with ME/CFS cared for in seven U.S. ME/CFS specialty clinics. The cognitive sub-study was designed with three primary objectives in mind. The first objective was to determine whether a brief cognitive battery, tailored to the ME/CFS cognitive deficiency profile as established in the peer-reviewed literature, (Sebaiti et al., 2022) reliably distinguishes between ME/CFS and HC over time. The second objective was to assess whether an all-day in-clinic study visit had a decremental effect on cognitive function. The third objective sought to determine whether a single short session of strenuous physical exercise would impact on cognitive function over and above that of the baseline clinical visit over 48 h comparing participants who did and did not undergo exercise testing (Cook et al., 2022).

## 2 Materials and methods

### 2.1 Study design

This MCAM cognitive sub-study was conducted between November 2013 and February 2019. Participants were recruited from the MCAM study and provided additional informed consent for their participation in the sub-study as approved by CDC’s Institutional Review Board (IRB), Western IRB for the Open Medicine Institute (OMI) consortium [covering Open Medicine Clinic (CA), Hunter Hopkins Clinic (NC), Richard Podell Clinic (NJ), Bateman Horne Center (UT), and Sierra Internal Medicine (NV)], Mount Sinai Beth Israel IRB for Pain & Fatigue Study Center (NY), and Nova Southeastern University IRB for Institute for Neuro Immune Medicine (FL).

### 2.2 Study sample

A total of 473 participants completed the baseline assessment of the cognitive sub-study. Of these, 47 were excluded from the analysis for the following reasons: (1) two withdrew from the study, one was later determined age ineligible; (2) four completed the CogState Brief Screening Battery (CBSB) but not two traditional neuropsychological measures [Wechsler Adult Intelligence Scale-4th Edition (WAIS-IV) digit span forward (DSF) and backward (DSB) (Wechsler, 2008)]; (3) 40 did not complete or had at least one failure on the completion and integrity check at T0 or T1 in-clinic sessions of the CBSB. Overall, ME/CFS patients were not excluded more than healthy controls (HC) (*n* = 32 (10.92%) vs. 15 (8.33%), *p* = 0.36). Thus, the sample included in the current analysis are 426 participants: 261 ME/CFS and 165 HC. Of 261 participants with ME/CFS, 182 also completed exercise testing.

### 2.3 Data source and workflow

The data collection workflow for this sub-study is summarized in Supplementary Table 1. The cognitive sub-study included a battery of questionnaires for assessing illness symptoms and functioning status, as well as traditional and computerized tasks for cognitive assessment. Computerized cognitive assessment was administered across five timepoints: two in-clinic assessments (T0, near the beginning of the clinic visit, before the exercise testing (if done) and T1 immediately after exercise or at end of clinic visit if no exercise testing) and up to three assessments administered at home (approximately 6–12 (T2), 24 (T3) and 48 h (T4) after participants’ clinic visit). A short practice battery, administered right before T0, familiarized participants with the visual task demands of the CBSB tasks; a study coordinator made certain that participants understood task demands. Once participants started the experimental CBSB at T0 and T1, the coordinator provided minimal assistance and typically waited in an adjoining room for participants to finish. All participants completed the Test of Premorbid Functioning (TOPF) only at T0 (Pearson, 2009). The traditional neurocognitive tests, WAIS-IV digit span forward (DSF) and backward (DSB), (Wechsler, 2008) often used for clinical screening of attention and working memory, were administered only in clinic (T0 and T1). All clinics with one exception, enrolled participants for standardized maximal cardiopulmonary exercise testing, ramped cycle ergometry to volitional exhaustion (Unger et al., 2017; Cook et al., 2022). The exercise testing, when performed, occurred between T0 and T1.

### 2.4 CogState brief screening battery (CBSB)

The CBSB includes practice items, culture free test stimuli (playing cards), and is considered to not be overly stressful or fatiguing by the majority of participants drawn from a large sample of the general population (Kuiper et al., 2017). Most of the measures included in this sub-study have been validated in illnesses that have non-focal, subtle brain involvement and objective cognitive symptom profiles similar to those documented in persons with ME/CFS [i.e., mild traumatic brain injury (Maruff et al., 2009)], have acceptable convergent, (Patel et al., 2017) and test-retest validity values (Kochan et al., 2022). When used for serial testing, small increases in scores are expected across time for both in clinic and remote administrations (Stricker et al., 2020). After the MCAM sub-study was initiated, the usefulness of CBSB in a large sample including patients with ME/CFS in the Netherlands was published, but only included four short computer tasks (Kuiper et al., 2017).

The CBSB developed and provided by CogState (CogState, Ltd, 2024) included a total of six computer tasks chosen for their relevance in detecting poor cognitive function associated with ME/CFS, if present. These tasks were chosen for measuring psychomotor speed/simple reaction time (Detection Task; DET), attention (Identification Task; IDN), recognition learning (One Card Learning Task; OCL), simple working memory (1-Back Task; ONB), complex working memory (2-Back Task; TWOB), and executive function/learning efficiency, complex problem solving under time constraints (Groton Maze Learning; GML). More details about CBSB can be found in Supplementary Table 2.

### 2.5 Neurocognitive outcome measures

The outcome variable for the non-timed DSF and DSB traditional neurocognitive tasks is number of verbally correctly recalled number sequences (higher score = better performance). We analyzed two types of outcome measures for the computerized CBSB tasks: 1) performance accuracy represented by the arcsine square root transformation of the proportion of correct responses (higher score = better performance), 2) performance speed represented by the mean of the log_10_-transformed reaction time (milliseconds) for correct responses (lower score = better performance), and for the GML task only, as probability of correct moves per second (higher score = better performance).

### 2.6 Statistical analysis

Participant characteristics were expressed as mean [standard deviations (SD)] for continuous variables and counts and percentages for categorical variables. We conducted chi-squared or t-tests to compare characteristics between two groups. The magnitude of each effect was reported as effect size, Cohen’s d with considering 0.2 as ‘small,’ 0.5 as ‘moderate’ and 0.8 as ‘large’. For cognitive test outcomes, we also used Cohen’s *d* > 0.3 reflecting clinical meaningfulness (Cohen, 1988; Joustra et al., 2022). To determine the interaction effects of time and case-control groups on CBSB outcome measures, repeated measures multivariate analysis of variance (MANOVA) was performed with the interaction between group and time. F statistics, *p*-values, and adjusted *p*-values by Greenhouse-Geisser and Huynh-Feldt-Lecoutre methods were reported for the interaction effect of time and groups. Spearman correlations were used to determine if the CBSB tasks converged with traditional neurocognitive tests in assessing cognitive function. We chose a two-sided significance threshold of *p* < 0.01, rather than a Bonferroni correction for multiple comparisons of CBSB outcomes, to guard against overly conservative corrections, which could increase the likelihood of Type II errors in this exploratory study. Bonferroni correction for the 12 outcome tests of significance would place the significance threshold at *p* ≤ 0.004 (=0.05/12). Socio-demographics such as age and sex, and pre-illness estimate of overall intellectual function (measured by TOPF) were used to adjust for the associations of the cognitive outcomes with study groups.

## 3 Results

Table 1 summarizes socio-demographic characteristics of the study sample (*n* = 426). Overall, participants had mean age of 47-years. The majority was female (69%), white (78%), non-Hispanic (85%), insured (93%), with a college or higher education attainment (73%). ME/CFS participants had 15-year mean duration of illness; 57% reported sudden onset. ME/CFS and HC were significantly different in age, race/ethnicity, and employment. As expected, ME/CFS participants had significantly worse illness symptoms and functioning than HC (see Supplementary Table 3, Cohen’s d = 0.8–3.3, all *p* < 0.0001).

**TABLE 1:** Socio-demographic Information of the Study Participants (*n* = 426).

Variables		ME/CFS (*n* = 261)		HC (*n* = 165)		Overall (*n* = 426)	
		*n*	%	*n*	%	*n*	%
Age****	18–29	25	9.58	41	24.85	66	15.49
	30–39	39	14.94	27	16.36	66	15.49
	40–49	49	18.77	33	20.00	82	19.25
	50–59	85	32.57	43	26.06	128	30.05
	>= 60	63	24.14	21	12.73	84	19.72
Sex	Male	77	29.50	54	32.73	131	30.75
	Female	184	70.50	111	67.27	295	69.25
Race****	White	223	90.28	82	49.70	305	78.01
	Black	6	2.43	18	10.91	24	6.14
	All others	18	7.29	44	26.67	62	15.86
Ethnicity****	Hispanic	13	5.35	45	27.27	58	14.61
	Non-Hispanic	230	94.65	109	66.06	339	85.39
Marital status	Married/committed	128	53.78	69	41.82	197	52.67
	Previously married	40	16.81	30	18.18	70	18.72
	Never married	70	29.41	37	22.42	107	28.61
Employment****	Full-time	36	16.00	83	50.30	119	33.52
	Part-time	24	10.67	24	14.55	48	13.52
	Not working	165	73.33	23	13.94	188	52.96
Insurance	Yes	230	95.04	122	73.94	352	92.63
	No	12	4.96	16	9.70	28	7.37
Education	Less than high school	2	0.80	3	1.82	5	1.22
	High school graduate	58	23.11	46	27.88	104	25.30
	College graduate	98	39.04	58	35.15	156	37.96
	Post college	93	37.05	53	32.12	146	35.52
Onset Status	Gradual	106	43.09	NA	NA	NA	NA
	Sudden	140	56.91	NA	NA	NA	NA
Illness Duration, years	Mean (SD)	15.24	10.23	NA	NA	NA	NA

**p-value < 0.01,

***p-value < 0.001,

****p-value < 0.0001; NA, Not Applicable. The frequency counts for missing values are not shown and so the total frequency counts (*n*) for some variables are not summed up to 261 ME/CFS or 165 HC.

### 3.1 Traditional neurocognitive tests

Table 2 summarizes the results of TOPF and WAIS-IV DSF and DSB tests between ME/CFS and HC. ME/CFS participants had higher TOPF standard scores than HC (116.5 vs. 111.8, *p* < 0.01, Cohen’s d = 0.4). No significant difference was found in age-corrected standard scores of DSF and DSB between groups at T0 and T1. Associations at T0 and T1 between the traditional tasks addressing attention and working memory often given by providers in clinic (DSF and DSB), and CBSB tasks were weak (see Supplementary Table 4).

**TABLE 2:** TOPF and WAIS-IV digit span tests.

		ME/CFS		HC		Difference	
Variable	Time	Mean	95% CI^a^	Mean	95% CI	ES d^b^	*p*-value
TOPF SS***	T0	116.49	(115.212, 117.767)	111.75	(109.585, 113.921)	0.406	0.0002
**WAIS-IV digit span test**							
DSF SS	T0	11.86	(11.471, 12.259)	11.22	(10.688, 11.755)	0.201	0.0514
DSF SS	T1	11.84	(11.404, 12.270)	11.50	(10.958, 12.036)	0.101	0.3305
DSB SS	T0	10.73	(10.375, 11.077)	10.23	(9.775, 10.680)	0.178	0.0840
DSB SS	T1	10.82	(10.406, 11.225)	10.90	(10.417, 11.379)	0.026	0.7981

^a^CI, Confidence Interval;

^b^ES, Effect Size, Cohen’s d = 0.2 be considered a “small” effect size, 0.5 represents a ‘moderate’ effect size and 0.8 a “large” effect size; TOPF, Test of Premorbid Functioning; DSF, Digit Span Forward (Attention); DSB, Digit Span Backward (Working Memory); SS = Age-corrected Standard Score or Scaled Score.

**p-value < 0.01,

***p-value < 0.001,

****p-value < 0.0001.

### 3.2 CogState brief screening battery (CBSB)

**FIGURE 1:**
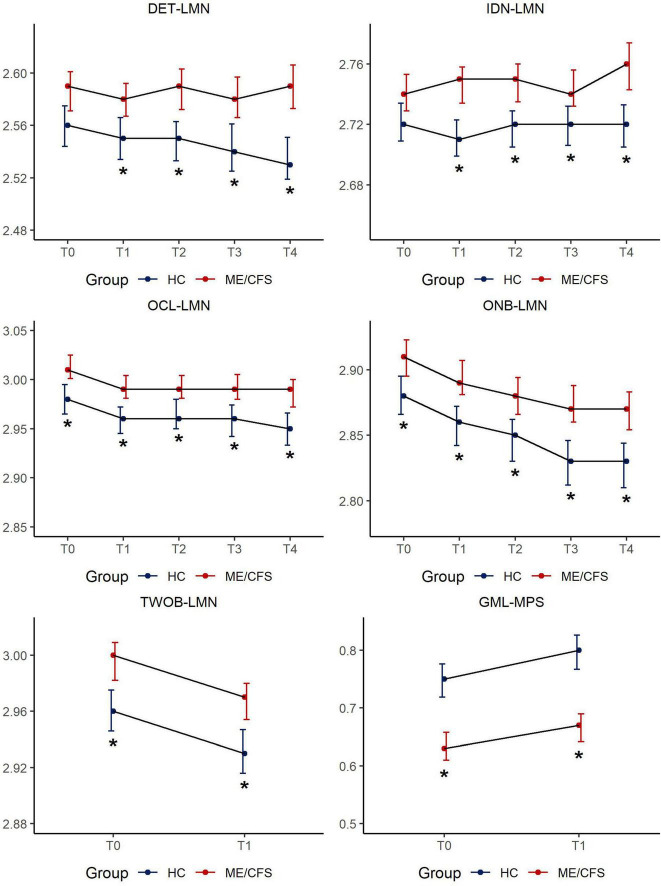
CBSB measures for speed of performance by study groups across timepoints. Estimated mean score [95% Confidence Interval (CI)] in CogState measures across 5 sessions: LMN = Speed is represented by the mean of the log10 transformed reaction times for correct responses; MPS = Moves per second; DET = Detection, IDN = Identification, OCL = One Card Learning, ONB = 1-Back, TWOB- 2-Back, GML = Groton Maze Learning; *p < 0.01.

Figure 1 depicts the results of CBSB performance speed over time and details of statistics are provided in Supplementary Tables 5a, b.

#### 3.2.1 Detection (DET-LMN for psychomotor speed)

There was no difference between ME/CFS and HC in latency to respond/simple reaction time at T0. The effect size for group mean differences in psychomotor speed/reaction time ranged from 0.3 to 0.5 (*p* < 0.01) over time reaching clinical meaningfulness (d > 0.3) at T2 (6–8 h after clinic visit) increasing in clinical meaningfulness (*d* = 0.5) at T4 (48-h post-clinic). Given the ME/CFS group’s significantly slower performance over time on DET-LMN, a task that mainly reflects the motoric aspect of responding to the visual stimulus, this variable was used as a covariate in the analysis of the remaining CBSB latency results to isolate the mental information processing time of study participants.

#### 3.2.2 Identification (IDN-LMN for attention)

There was no difference between ME/CFS and HC in response latency at T0. Participants with ME/CFS showed a variable, but largely increased response time on this choice reaction time task, while HCs maintained a steady level of attention over time. The effect size for group mean differences on this simple attention task ranged from 0.2 to 0.4 (*p* < 0.01) over time.

#### 3.2.3 One card learning and one-back working memory tasks (OCL-LMN for learning/memory, ONB-LMN for simple working memory)

These cognitive tasks are more complex, involve simple learning and working memory components, calling for minor mental multi-tasking. Compared to HCs, it took longer for ME/CFS participants to respond to task challenges across all timepoints (OCL-LMN: Cohen’s d = 0.3–0.4, all *p* < 0.01, ONB-LMN: *d* = 0.3–0.5, all *p* < 0.01). Despite significant differences in information processing latencies, both groups’s response latency improved with task repetition. There were no significant group by time interaction effects on either DET-LMN, IDN-LMN, OCL-LMN, or ONB-LMN (all *p* > 0.05, Supplementary Table 5b).

#### 3.2.4 Two-back working memory task (TWOB-LMN for complex working memory), groton maze learning task (GML-MPS for executive function/learning efficiency)

Both TWOB and GML tasks reflect increased cognitive complexity tapping into executive function and learning efficiency and necessitating a greater degree of mental multitasking. Both tasks were only administered at T0 and T1 in clinic as a remote administration option for these tasks had not been developed at the time of study enrollment. Compared to HCs, it took longer for ME/CFS participants to respond to either task at both T0 and T1. Group mean differences were clinically meaningful to a small degree for TWOB-LMN (d = 0.3 for both timepoints), and to a moderate degree for GML-MPS, (T0: d = 0.6; T1: d = 0.7).

The results of CBSB performance accuracy outcomes are provided in Supplementary Figure 1 and Supplementary Table 6. With the exception of a significant finding of slowed reaction time affecting performance accuracy for the Detection task in ME/CFS participants at T4 (d = 0.3), there were no differences in performance accuracy between ME/CFS and HC on any of the six tasks over time.

### 3.3 Impact of exercise testing on cognitive function

We sought to evaluate whether the exercise testing produced a differential impact on CBSB performance over time over and above the efforts associated with the clinical visit that included cognitive testing (“exercise” vs. “no-exercise”).

Table 3 summarizes latency responses across timepoints between “exercise” and “no-exercise” ME/CFS participants. The “no-exercise” group did not differ from the “exercise” group in response latency on any CBSB tasks except for GML (GML-MPS: (T0: 56% versus 66%, d = 0.5; T1: 59% versus 70%, d = 0.6; all *p* < 0.001). Significant differences were found in race and education between “exercise” and “no-exercise” groups but not in any measures for overall functioning and illness symptoms, prompting us to also adjust the comparison for race and education in addition to age, TOPF performance, and psychomotor speed. With the adjustment of age, race, education, TOPF, and psychomotor speed, the GML-MPS differences remained significant. While group differences were found for GML-MPS at both timepoints, with the ME/CFS group completing an exercise component showing better executive function/learning efficiency, the exercise testing did not contribute to differences in the practice effect observed from T0 to T1 (Stricker et al., 2020).

**TABLE 3:** Latency to respond between ME/CFS participants with and without exercise testing across timepoints (*n* = 261).

		Clinical visit (*n* = 79)		Clinical visit + exercise (*n* = 182)		Difference	
Variable	Time	Mean	95% CI^a^	Mean	95% CI	ES d^b^	*p*-value
DET-LMN	T0	2.61	(2.579, 2.644)	2.57	(2.559, 2.590)	0.313	0.0426
DET-LMN	T1	2.58	(2.556, 2.607)	2.58	(2.564, 2.594)	0.023	0.8671
DET-LMN	T2	2.60	(2.567, 2.638)	2.58	(2.566, 2.600)	0.183	0.2852
DET-LMN	T3	2.60	(2.558, 2.633)	2.58	(2.561, 2.593)	0.158	0.3770
DET-LMN	T4	2.59	(2.551, 2.622)	2.59	(2.572, 2.609)	0.035	0.8235
IDN-LMN	T0	2.75	(2.724, 2.780)	2.74	(2.723, 2.749)	0.160	0.3050
IDN-LMN	T1	2.74	(2.715, 2.758)	2.75	(2.736, 2.765)	0.150	0.2717
IDN-LMN	T2	2.75	(2.720, 2.784)	2.75	(2.732, 2.760)	0.069	0.6890
IDN-LMN	T3	2.73	(2.708, 2.758)	2.75	(2.735, 2.763)	0.180	0.2351
IDN-LMN	T4	2.75	(2.716, 2.786)	2.76	(2.744, 2.778)	0.084	0.6269
OCL-LMN	T0	3.01	(2.987, 3.032)	3.01	(3.001, 3.028)	0.057	0.6792
OCL-LMN	T1	2.98	(2.963, 3.007)	3.00	(2.982, 3.009)	0.121	0.3793
OCL-LMN	T2	2.99	(2.962, 3.010)	2.99	(2.981, 3.008)	0.105	0.5352
OCL-LMN	T3	2.98	(2.957, 3.009)	3.00	(2.982, 3.011)	0.141	0.3560
OCL-LMN	T4	2.98	(2.948, 3.010)	2.99	(2.974, 3.004)	0.099	0.5761
ONB-LMN	T0	2.91	(2.885, 2.940)	2.91	(2.892, 2.923)	0.042	0.7641
ONB-LMN	T1	2.89	(2.863, 2.912)	2.90	(2.882, 2.913)	0.094	0.5016
ONB-LMN	T2	2.88	(2.846, 2.910)	2.88	(2.865, 2.896)	0.026	0.8774
ONB-LMN	T3	2.85	(2.824, 2.878)	2.88	(2.867, 2.899)	0.318	0.0378
ONB-LMN	T4	2.85	(2.821, 2.884)	2.87	(2.858, 2.891)	0.205	0.1898
TWOB-LMN	T0	2.99	(2.968, 3.020)	3.00	(2.981, 3.012)	0.030	0.8277
TWOB-LMN	T1	2.95	(2.931, 2.974)	2.97	(2.957, 2.989)	0.193	0.1643
GML-MPS****	T0	0.56	(0.519, 0.602)	0.66	(0.636, 0.693)	0.546	0.0001
GML-MPS****	T1	0.59	(0.554, 0.633)	0.70	(0.669, 0.727)	0.555	0.0001

^a^CI, Confidence Interval;

^b^ES, Effect Size, Cohen’s d = 0.2 be considered a ‘small’ effect size, 0.5 represents a ‘moderate’ effect size and 0.8 a ‘large’ effect size; LMN = Speed is represented by the mean of the log10 transformed reaction times for correct responses; MPS = Moves per second; DET = Detection (Psychomotor Speed), IDN = Identification (Attention), OCL = One Card Learning (Learning and Memory), ONB = 1-Back, TWOB- 2-Back (Working Memory), GML = Groton Maze Learning (Executive Function).

**p-value < 0.01,

***p-value < 0.001,

****p-value < 0.0001.

## 4 Discussion

Our findings show that CBSB is sensitive to detect the presence of the established cognitive symptom profile of ME/CFS (Sebaiti et al., 2022) in clinic and can be used remotely, as a self-administered screening battery, to monitor changes in cognitive function over time. This MCAM cognitive sub-study showed that persons with ME/CFS are able to react and attend to simple cognitive tasks as well as HCs at the outset of the study visit (T0), but are not able to maintain these challenges over time. In fact, while their simple reaction time remains unchanged simple attention becomes more variable over 48 h leading to poorer task performance compared to HCs. In contrast, on tasks requiring increased cognitive efficiency and “multitasking” involving learning, memory, working memory, and executive function, significant differences in response latency or information processing speed (Sweet, 2011), are already evident at T0, in clinic, and increase over time to clinically meaningful degrees. The use of accuracy as an outcome measure did not prove to be sensitive enough to determine ME/CFS cognitive dysfunction. Performance accuracy of ME/CFS participants was similar to that of HCs across timepoints and tasks. In contrast, performance latency/speed of mental information processing objectively determined the presence and nature of cognitive dysfunction experienced by persons with ME/CFS in clinic and up to 48-h later at home. Our data show the addition of a maximal exercise test to the study visit did not result in further cognitive dysfunction above and beyond that provoked by the clinical visit. Cvejic and colleagues (Cvejic et al., 2016) provided a useful framework to conceptualize cognitive dysfunction in ME/CFS presenting with adequate performance accuracy but neural inefficiency reflected by slower processing speed observed especially on tasks requiring complex information processing, decision-making, and efficient learning of new information. Our data show that “traditional” simple attention and working memory tasks, developed to assess accuracy of performance, and often used in clinic and research, do not detect the subtle cognitive symptom profile in ME/CFS. Thus, if used as the sole screening tool for cognitive dysfunction in ME/CFS, their usefulness is limited as they will not capture the hallmark of cognitive dysfunction in ME/CFS that is reflected by a compromised ability to effectively and efficiently execute tasks of daily life that can include trying out a new recipe or developing a new marketing strategy.

Joustra et al. (2022) administered the online CogState battery comprised of four tasks (DET-LMN, IDN-LMN, OCL-ACC, OBK-LMN) during an onsite study visit to a large population-based sample that included 70,951 healthy controls and 2461 participants with ME/CFS fulfilling the 1994 research case definition as assessed with symptom questionnaires and physical examination. They found that participants with ME/CFS performed significantly poorer on all tasks compared to healthy controls though with a small effect size. They did not include a correction for motor reaction time (DET-LMN) and education. The length of time of the study visit and timing of the cognitive test relative to other steps of the study visit are not specified and may explain the small effect size. Our findings of larger effect sizes after a full day study visit (T0 at start and T1 at end) with further differences in follow-up indicate the importance of repeat and at home testing.

Our findings add to the growing realization that persons with ME/CFS are compromised cognitively, they are reacting slower, attention is variable over time, and cognitive efficiency supporting executive function, learning and memory is significantly decreased to a clinically meaningful degree. The adoption of remote cognitive screening over time shows that cognitive dysfunction is and remains present after a clinical visit with or without stressful physical activity.

Protocolized repeatable cognitive screenings have long been devised and implemented for disorders with anatomical brain illness markers such as Multiple Sclerosis i.e., (Benedict et al., 2012) and Epilepsy i.e., (Kurzbuch et al., 2013). However, this development has been lacking for disorders without focal, observable brain involvement including ME/CFS. In our opinion, this is a significant disservice to these patient groups. It is essential to compare results of cognitive screenings across studies in a more reliable, standardized, and valid way than is currently done. Efforts to do so are ongoing by the ME/CFS Common Data Elements (CDE) consortium spearheaded by NIH and CDC. While that is an important step forward for research, brief, repeatable, computerized, and cost-effective screening tools need to be available for clinicians, not necessarily trained in neuropsychological assessment, to quickly make an initial diagnosis about whether or not cognitive decrements in their ME/CFS patients are present and changing over time to optimally manage illness symptoms and quality of life in ME/CFS patients.

Our study has limitations. First, the study sample came from U.S. ME/CFS specialty clinics providing us with a diverse and large sample of well-characterized ME/CFS patient populations with geographic representation. This may limit the generalizability of our findings to patients in primary care populations. Second, TWOB and GML tasks were not administered remotely. Thus, we were not able to determine whether exercise would impact performance speed on complex working memory and executive function/learning efficiency tasks after the clinic visit (approximately 6–12, 24, and 48 h after the exercise testing). Third, the comparison addressing the cognitive impact of the clinic visit with or without exercise testing was only performed among patients with ME/CFS (see Table 3) as few HCs (5 out of 165) participated in the cognition only group (i.e. the clinical visit without exercise). Thus, we cannot compare physical deterioration with regard to HCs, but only within the group of ME/CFS participants. Fourth, while cognitive function in ME/CFS Black and Latino populations may be more affected, small sample sizes for these racial/ethnic groups (Black: *n* = 6; Hispanic: *n* = 13) prevented us from performing a meaningful analysis to further explore this issue. Lastly, lack of verbal tasks limits our findings to the visual cognitive domain. Future studies including verbal tasks may help determine ME/CFS cognitive dysfunction in verbally mediated cognitive tasks over time.

## 5 Conclusion

The CBSB is sensitive to detect objective deficits in cognitive function in persons with ME/CFS in clinic and remotely over time when speed of performance is used as an outcome measure. The physical exertion of a single maximal cardiopulmonary exercise test does not further exacerbate the magnitude of cognitive deficit over time.

## Data Availability

The datasets presented in this article are not readily available because Restrictions by the data custodians mean that the datasets are not publicly available or able to be provided by the authors. The program codes used in the current study are available from the corresponding author on reasonable request. Researchers wanting to access the datasets used in this study should email CDC’s ME/CFS Program (cfs@cdc.gov) and discuss next steps for the data request. The ME/CFS program data review committee will grant the access after the review and the data use agreement is finalized. Requests to access the datasets should be directed to cfs@cdc.gov.
