# Supplementary figures and images for: Cognitive assessment in myalgic encephalomyelitis/chronic fatigue syndrome (ME/CFS): a cognitive substudy of the multi-site clinical assessment of ME/CFS (MCAM)
Source: Front Neurosci. 2024 Nov 1;18:1460157. doi: 10.3389/fnins.2024.1460157 (PMC11565701; doi:10.3389/fnins.2024.1460157)

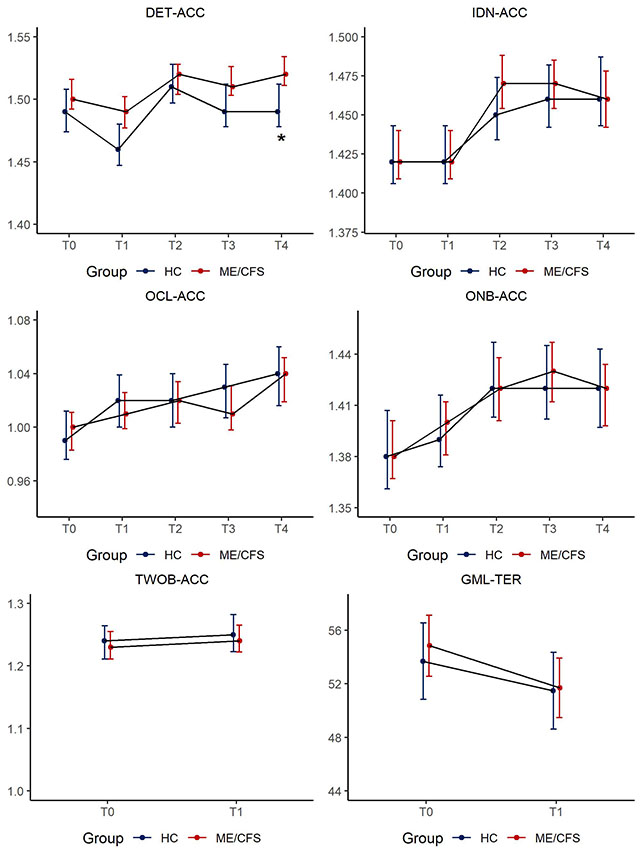

Supplement: Supplementary file 1 [file Image_1.jpeg]
