# Supplementary material for: Cognitive assessment in myalgic encephalomyelitis/chronic fatigue syndrome (ME/CFS): a cognitive substudy of the multi-site clinical assessment of ME/CFS (MCAM)
Source: Front Neurosci. 2024 Nov 1;18:1460157. doi: 10.3389/fnins.2024.1460157 (PMC11565701; doi:10.3389/fnins.2024.1460157)
Supplement: Supplementary file 2 [file Data_Sheet_1.docx]

**SUPPLEMENT 1: SUPPLEMENTAL ONLINE CONTENT**

**eTable 1.** Tasks/Data Collection Workflow for the Cognitive Sub-study

**eTable 2.** Detailed Description of Neuropsychological Tasks Administered

**eTable 3.** Overall Functioning and Symptom Profile by Study Groups

**eTable 4.** Spearman Correlations between Cognitive Domains Assessed by CBSB and Traditional Neuropsychological Tests (WAIS-IV Digit Span)

**eTable 5a.** CBSB Measures for Speed of Performance by Study Groups over Timepoints

**eTable 5b.** Repeated measured MANOVA test between subjects and within subjects for CBSB Speed of Performance Variables over Timepoints

**eFigure 1.** CBSB Measures for Accuracy of Performance by Study Groups across Timepoints

**eTable 6.** CBSB Measures for Accuracy of Performance by Study Groups

**eReferences**

This supplemental material has been provided by the authors to give readers additional information about their work.

**eTable 1.** **Tasks/Data Collection Workflow** **for** **the Cognitive Sub-study**

| **Time** | **Tasks/Data Collection** |
| --- | --- |
| **At Clinic** |  |
| Check-in | Admission and Enrollment – consent form |
| After the admission | Basic Evaluation : Self-reported Questionnaires : ~10-15 min  1) VAS- ME/CFS Symptoms #1, 2) EQ-5D #1, 3) PROMIS SF–PF 12a, 4) PFE*, 5) PAR-Q, 6) IPAQ* |
| Practice (AM in clinic) | CBSB Practice Session on Six Cognitive Tests: ~17 minutes  1) Detection (DET), 2) Identification (IDN), 3) One Card Learning (OCL), 4) 1- Back (ONB) and 5) 2-Back Working Memory (TWOB), 6) Groton Maze Learning (GML) |
| T0 (AM in clinic) | Complete Eight Cognitive Tests: ~27 minutes   - CBSB tasks: 1) DET, 2) IDN), 3) OCL, 4) ONB and 5) 2-Back Working Memory (TWOB), 6) GML - Traditional tests: 1) WAIS-IV DSF + DSB, 2) TOPF |
|  | **Exercise Session: 1-2 hours between AM clinic and PM clinic** |
| T1 (PM in clinic) | Complete Seven Cognitive Tests: ~22 minutes   - CBSB tasks: 1) DET, 2) IDN), 3) OCL, 4) ONB, 5) TWOB, 6) GML tasks - Traditional tests: WAIS IV DSF + DSB |
| **After Clinic Visit (Remotely At Home)** |  |
| T2 (6~12 hours after the admission) | Complete Four CBSB Tests (remote): ~12 minutes  CBSB tasks: 1) DET, 2) IDN), 3) OCL, 4) ONB |
|  | Questionnaire (~3 min) : VAS- ME/CFS Symptoms #2 |
| T3 (~24 hours after the admission) | Complete Four CogState Tests (remote): ~12 minutes  CBSB tasks: 1) DET, 2) IDN), 3) OCL, 4) ONB |
|  | Questionnaire (~5 min) : 1) VAS- ME/CFS Symptoms #3, 2) EQ-5D #2 |
| T4 (~48 hours after the admission) | Complete Four CogState Tests (remote): ~12 minutes  CBSB tasks: 1) DET, 2) IDN), 3) OCL, 4) ONB |
|  | Questionnaire (~5 min) : 1) VAS- ME/CFS Symptoms #4, 2) EQ-5D #3 |
| T5 (~96 hours after the admission) | Questionnaire (~5 min) : 1) VAS- ME/CFS Symptoms #5, 2) EQ-5D #4 |
|  |  |

VAS= Visual Analogue Scale (Aitken, 1969); EQ-5D= Europe Quality of Life (Euroqol, 2014); PROMIS SF–PF 12a= Patient Reported Outcome Measurement Information System (PROMIS) Physical Function Short Form 12a (Rose et al., 2008); PFE= Physical Fitness and Exercise (Devereaux Melillo et al., 1997); PAR-Q= Physical Activity Readiness Questionnaire (British Columbia Ministry of Health, 1978; Thomas et al., 1992); IPAQ= International Physical Activity Questionnaire (Booth, 2000; Craig et al., 2003); CBSB= CogState Brief Screening Battery (CogState, LTD.); WAIS-IV = Wechsler Adult Intelligence Scale Fourth Edition); DSF= Digit Span Forward; DSB = Digit Span Backward (Wechsler, 2008; TOPF= Test of Premorbid Functioning (Pearson, 2009); *indicating the instruments not administered to those enrolled for the Cognition testing only.

**eTable 2: Detailed Description of Neuropsychological Tasks Administered**

| **CogState Brief Screening Battery (CBSB)** | |
| --- | --- |
| Detection Task (DET): Task stimuli are images of playing cards showing either red or black jokers. The DET measures simple reaction time (RT) defined as the time it takes to press a “Yes” button as soon as a playing card in the center of the screen flips over. |  |
| Identification Task (IDN): Task stimuli are again images of playing cards showing either red or black jokers. The IDN task measures attention by employing a *choice* reaction time paradigm. Participants have to indicate whether or not a playing card is red as quickly as possible by pressing “Yes” if the playing card is red and “No” if it is black. |  |
| One Card Learning Task (OCL): Task stimuli are again playing cards, but this time without jokers. The OCL task measures recognition memory by employing a *pattern separation* paradigm. Participants press the “Yes” or “No” button to indicate whether or not they recognize having seen the currently displayed card at any time previously in the deck. |  |
| 1-Back Working Memory Task (ONB): Task stimuli again are playing cards without jokers. The ONB measure working memory by employing the *n-back* paradigm. Again, participants press a “Yes” or “No” button as quickly as possible to indicate whether or not the current card presented is the same as *the card just previously presented* (one back) or not. |  |
| 2-Back Working Memory Task (TWOB): Task stimuli again are playing cards without jokers. The TWOB measures working memory also by employing the *n-back* paradigm. In the TWOB task, participants press the “Yes” or “No” button as quickly as possible to indicate whether or not the current card presented is the same as *the card shown two cards previously* (two back). The first response is always “No” because no comparison card has been presented yet. |  |
| Groton Maze Learning Task (GML): The GML assesses problem solving, reasoning, and efficient learning under time pressure using a *maze learning* paradigm. A 28-step pathway is hidden among 100 possible locations in a 10 × 10 grid of tiles on the screen. Each box represents move locations, and the grid refers to the box array (i.e., 10 × 10). Participants are required to find a hidden pathway guided by four search rules. These rules are: 1) do not move diagonally, 2) do not move more than one box (i.e., do not jump), 3) do not move back on the pathway, and 4) return to the last correct location after an error. Feedback is given with visual and auditory cues (green check marks and red crosses as well as beeps) to indicate whether the selected box is correct or incorrect. The last correct location, flashes with a green check when two errors are made in succession (failing to return errors). At each step only the most recently selected box is shown. There are 20 well-matched alternate pathways available. The software records each move as either an error or as a correct move. The primary outcomes are the total number of errors made in attempting to learn the same hidden pathway on five consecutive trials at a single session (GML-TER) and the total number of correct moves made per second (GML-MPS). |  |
| **Traditional Neuropsychological Tests** |  |
| The following two traditional neuropsychological tests were used and took about 10 minutes to be completed. |  |
| Test of Premorbid Functioning (TOPF (Pearson, 2009): The TOPF requires participants to read a list of 70 phonetically irregular words. The ability to successfully pronounce irregularly spelled words is relatively resistant to neurological injury and a sensitive marker of intellectual attainment (Holdnack, et al., 2013). Participants must read and pronounce a list of words printed in two columns on the front and back of a card. The TOPF was scored according to procedures outlined in the manual (Pearson, 2009). The best possible raw score for the TOPF is 70 and a derived age-corrected standard score (SS) can be used to predict the expected premorbid WAIS-IV Full Scale IQ score. The TOPF was only administered at T0. |  |
| The Wechsler Adult Intelligence Scale, Fourth Edition (WAIS-IV) Digit Span Forward and Backward tasks (DSF & DSB) (Wechsler, 2008): These two tasks were verbally administered. The DSF is often described as a test of simple auditory attention while the DSB is a test of simple auditory working memory. In the DSF test, participants are asked to repeat digits, the length of the digit sequence is increased across trials until there is a failure across two consecutive trials of a particular length. In the DSB test, participants are asked to repeat digits backwards across trials until there is a failure across two consecutive trials of a particular length. Raw scores range from 0 to 16 on both tasks and can be converted to age-corrected Standard Scores (SS). DSF and DSB were only administered at T0 and T1. |  |

**eTable 3: Overall Functioning and Symptom Profile by Study Groups**

|  | ME/CFS (n=261) | | HC (n=165) | | Difference | |
| --- | --- | --- | --- | --- | --- | --- |
| Variable | Mean | 95% CI^a^ | Mean | 95% CI | ES d^b^ | p-value |
| **SF-36 T-scores** |  |  |  |  |  |  |
| Physical Component Summary | 28.39 | (27.100,29.688) | 55.43 | (54.521,56.333) | 3.054 | 0.0000 |
| Mental Component Summary | 41.42 | (39.862,42.968) | 52.84 | (51.521,54.151) | 1.036 | 0.0000 |
| Physical Functioning | 33.79 | (32.554,35.029) | 54.34 | (53.467,55.219) | 2.413 | 0.0000 |
| Role Physical | 26.11 | (24.917,27.310) | 53.93 | (52.848,55.006) | 3.214 | 0.0000 |
| Bodily Pain | 37.73 | (36.385,39.085) | 56.53 | (55.361,57.697) | 1.941 | 0.0000 |
| Vitality | 31.65 | (30.517,32.779) | 56.60 | (55.158,58.034) | 2.739 | 0.0000 |
| General Health | 30.05 | (28.962,31.138) | 54.82 | (53.542,56.107) | 2.905 | 0.0000 |
| Role Emotional | 41.73 | (39.774,43.684) | 52.70 | (51.534,53.867) | 0.836 | 0.0000 |
| Social Functioning | 27.30 | (25.879,28.724) | 52.22 | (51.002,53.435) | 2.458 | 0.0000 |
| Mental Health | 44.56 | (43.147,45.970) | 53.40 | (51.994,54.811) | 0.844 | 0.0000 |
| **MFI-20** |  |  |  |  |  |  |
| General Fatigue | 17.58 | (17.225,17.932) | 8.34 | (7.753,8.926) | 2.888 | 0.0000 |
| Physical Fatigue | 16.83 | (16.419,17.246) | 6.85 | (6.368,7.337) | 3.091 | 0.0000 |
| Reduced Activity | 15.85 | (15.375,16.331) | 7.19 | (6.690,7.689) | 2.412 | 0.0000 |
| Reduced Motivation | 11.38 | (10.887,11.879) | 7.26 | (6.770,7.748) | 1.128 | 0.0000 |
| Mental Fatigue | 14.54 | (14.046,15.043) | 7.78 | (7.210,8.351) | 1.762 | 0.0000 |
| **CDC-SI** |  |  |  |  |  |  |
| # of CFS Symptoms | 5.84 | (5.581,6.104) | 0.55 | (0.375,0.719) | 3.265 | 0.0000 |
| Summary of CFS Symptom Score | 51.80 | (48.495,55.095) | 2.72 | (1.630,3.809) | 2.572 | 0.0000 |
| **PROMIS T-scores** |  |  |  |  |  |  |
| Fatigue | 66.38 | (65.417,67.338) | 43.92 | (42.651,45.184) | 2.855 | 0.0000 |
| Sleep Disturbance | 59.10 | (58.110,60.085) | 45.94 | (44.534,47.355) | 1.574 | 0.0000 |
| Sleep-Related Impairment | 61.78 | (60.734,62.830) | 43.47 | (41.870,45.067) | 2.001 | 0.0000 |
| Pain Intensity | 60.53 | (59.336,61.734) | 44.76 | (43.726,45.796) | 1.838 | 0.0000 |
| Pain Behavior | 56.58 | (55.597,57.554) | 42.74 | (41.340,44.134) | 1.684 | 0.0000 |

^a^CI= Confidence Interval; ^b^ES= Effect Size, Cohen’s d = 0.2 be considered a 'small' effect size, 0.5 represents a 'moderate' effect size and 0.8 a 'large' effect size; SF-36= 36-item Short Form (Ware and Sherbourne, 1992; Ware, 2000)^;^ MFI=20= 20-item Multidimensional Fatigue Inventory (Smets et al., 1995); CDC-SI= CDC Symptom Inventory (Wagner et al., 2005); PROMIS= Patient-Reported Outcome Measurement System (Cella et al., 2007); The measures included in this table were collected from the main study (Unger 2017) not part of the Cognitive and Exercise Sub-study summarized in eTable 1.

**eTable 4: Spearman Correlations between Cognitive Domains Assessed by CBSB and Traditional Neuropsychological Tests (WAIS-IV Digit Span)**

|  |  | Traditional Neuropsychologic Tests | | | |
| --- | --- | --- | --- | --- | --- |
|  |  | T0 | | T1 | |
|  | CBSB | DSF SS  (Attention) | DSB SS  (Working Memory) | DSF SS  (Attention) | DSB SS  (Working Memory) |
| T0 | DET-ACC  (Psychomotor Speed) | **-0.203**  **(p<.0001)** | **-0.196**  **(p=0.0001)** |  |  |
| T0 | IDN-ACC  (Attention) | **-0.168**  **(p=0.0010)** | **-0.209**  **(p<.0001)** |  |  |
| T0 | OCL-ACC  (Learning and Memory) | -0.067  (p=0.2025) | 0.004  (p=0.9330) |  |  |
| T0 | ONB-ACC  (Simple Working Memory) | **-0.150**  **(p=0.0043)** | **-0.193**  **(p=0.0002)** |  |  |
| T0 | TWOB-ACC  (Complex Working Memory) | -0.035  (p=0.4987) | -0.041  (p=0.4246) |  |  |
| T0 | GML-TER  (Executive Function) | 0.045  (p=0.3759) | 0.031  (p=0.5372) |  |  |
| T1 | DET-ACC  (Psychomotor Speed) |  |  | **-0.212**  **(p<.0001)** | **-0.310**  **(p<.0001)** |
| T1 | IDN-ACC  (Attention) |  |  | **-0.159**  **(p=0.0019)** | **-0.221**  **(p<.0001)** |
| T1 | OCL-ACC  (Learning and Memory) |  |  | -0.065  (p=0.2178) | -0.015  (p=0.7824) |
| T1 | ONB-ACC  (Simple Working Memory) |  |  | **-0.109**  **(p=0.0404)** | **-0.155**  **(p=0.0034)** |
| T1 | TWOB-ACC  (Complex Working Memory) |  |  | -0.102  (p=0.0509) | **-0.120**  **(p=0.0213)** |
| T1 | GML-TER  (Executive Function) |  |  | 0.082  (p=0.1109) | **0.156**  **(p=0.0023)** |

ACC= Accuracy is represented by the arcsine transformation of the square root of the proportion of correct responses; TER= Total number of errors; DET= Detection (Psychomotor Speed), IDN= Identification (Attention), OCL= One Card Learning (Learning and Memory), ONB= 1-Back, TWOB= 2-Back (Working Memory), GML= Groton Maze Learning (Executive Function); Digit Span Forward (Attention)= DSF; Digit Span Backward (Working Memory)= DSB; Age-corrected Standard Score or Scaled Score= SS

**eTable 5a. CBSB Measures for Speed of Performance by Study Groups over Timepoints**

|  |  | ME/CFS (n=261) | | HC (n=165) | | Difference | |
| --- | --- | --- | --- | --- | --- | --- | --- |
| Variable | Time | Mean | 95% CI^a^ | Mean | 95% CI | ES d^b^ | p-value |
| DET-LMN | T0 | 2.59 | (2.571,2.601) | 2.56 | (2.544,2.575) | 0.239 | 0.0146 |
| DET-LMN | T1* | 2.58 | (2.567,2.592) | 2.55 | (2.534,2.566) | 0.297 | **0.0036** |
| DET-LMN | T2* | 2.59 | (2.572,2.603) | 2.55 | (2.533,2.563) | 0.396 | **0.0003** |
| DET-LMN | T3* | 2.58 | (2.566,2.597) | 2.54 | (2.525,2.561) | 0.347 | **0.0019** |
| DET-LMN | T4* | 2.59 | (2.573,2.606) | 2.53 | (2.519,2.551) | 0.493 | **0.0000** |
| IDN-LMN | T0 | 2.74 | (2.729,2.753) | 2.72 | (2.709,2.734) | 0.212 | 0.0266 |
| IDN-LMN | T1* | 2.75 | (2.734,2.758) | 2.71 | (2.699,2.723) | 0.389 | **0.0001** |
| IDN-LMN | T2* | 2.75 | (2.735,2.760) | 2.72 | (2.705,2.729) | 0.369 | **0.0007** |
| IDN-LMN | T3* | 2.74 | (2.732,2.756) | 2.72 | (2.706,2.732) | 0.306 | **0.0041** |
| IDN-LMN | T4* | 2.76 | (2.743,2.774) | 2.72 | (2.705,2.733) | 0.386 | **0.0002** |
| OCL-LMN | T0* | 3.01 | (3.001,3.025) | 2.98 | (2.965,2.995) | 0.351 | **0.0007** |
| OCL-LMN | T1* | 2.99 | (2.981,3.004) | 2.96 | (2.945,2.972) | 0.384 | **0.0002** |
| OCL-LMN | T2* | 2.99 | (2.981,3.004) | 2.96 | (2.950,2.980) | 0.330 | **0.0044** |
| OCL-LMN | T3* | 2.99 | (2.980,3.005) | 2.96 | (2.942,2.974) | 0.375 | **0.0010** |
| OCL-LMN | T4* | 2.99 | (2.972,3.000) | 2.95 | (2.933,2.966) | 0.384 | **0.0009** |
| ONB-LMN | T0* | 2.91 | (2.895,2.923) | 2.88 | (2.866,2.895) | 0.286 | **0.0005** |
| ONB-LMN | T1* | 2.89 | (2.881,2.907) | 2.86 | (2.842,2.872) | 0.382 | **0.0003** |
| ONB-LMN | T2* | 2.88 | (2.866,2.894) | 2.85 | (2.830,2.862) | 0.361 | **0.0018** |
| ONB-LMN | T3* | 2.87 | (2.860,2.888) | 2.83 | (2.812,2.846) | **0.457** | **0.0001** |
| ONB-LMN | T4* | 2.87 | (2.854,2.883) | 2.83 | (2.810,2.844) | 0.405 | **0.0004** |
| TWOB-LMN | T0* | 3.00 | (2.982,3.009) | 2.96 | (2.946,2.975) | 0.343 | **0.0008** |
| TWOB-LMN | T1* | 2.97 | (2.954,2.980) | 2.93 | (2.916,2.947) | 0.344 | **0.0009** |
| GML-MPS | T0* | 0.63 | (0.610,0.658) | 0.75 | (0.719,0.776) | **0.594** | **0.0000** |
| GML-MPS | T1* | 0.67 | (0.642,0.690) | 0.80 | (0.767,0.826) | **0.680** | **0.0000** |

^a^CI= Confidence Interval; ^b^ES= Effect Size, Cohen’s d = 0.2 be considered a 'small' effect size, 0.5 represents a 'moderate' effect size and 0.8 a 'large' effect size; LMN= Speed is represented by the mean of the log10 transformed reaction times for correct responses (lower score= better performance); MPS= Moves per second; DET= Detection, IDN= Identification, OCL= One Card Learning, ONB= 1-Back, TWOB= 2-Back, GML= Groton Maze Learning (higher score= better performance)

* p-value< 0.01 after adjusting for age

**eTable 5b.** Repeated measured MANOVA test between subjects and within subjects for CBSB Speed of Performance Variables over Timepoints

| **Variable** | **Source** | **DF** | **Type III** | **Mean** | **F** | **p-value** | **Adjusted p-value** | |
| --- | --- | --- | --- | --- | --- | --- | --- | --- |
|  |  |  | **SS** | **Square** | **Value** |  | **G-G** | **H-F-L** |
| **Between Subjects** | | | | | | | | |
| DET-LMN | Group | 1 | 0.5484 | 0.5484 | 14.5000 | 0.0002 |  |  |
| IDN-LMN | Group | 1 | 0.3109 | 0.3109 | 11.7200 | 0.0007 |  |  |
| OCL-LMN | Group | 1 | 0.2881 | 0.2881 | 9.3000 | 0.0025 |  |  |
| ONB-LMN | Group | 1 | 0.4117 | 0.4117 | 11.6100 | 0.0008 |  |  |
| TWOB-LMN | Group | 1 | 0.2181 | 0.2181 | 11.6700 | 0.0007 |  |  |
| GML-MPS | Group | 1 | 2.8043 | 2.8043 | 42.6200 | <.0001 |  |  |
| **Within Subjects** | | | | | | | | |
| DET-LMN | Time | 4 | 0.0173 | 0.0043 | 1.4100 | 0.2282 | 0.2342 | 0.2337 |
| DET-LMN | Group*Time | 4 | 0.0228 | 0.0057 | 1.8600 | 0.1148 | 0.1257 | 0.1247 |
| IDN-LMN | Time | 4 | 0.0488 | 0.0122 | 5.3400 | 0.0003 | 0.0006 | 0.0005 |
| IDN-LMN | Group*Time | 4 | 0.0127 | 0.0032 | 1.3900 | 0.2336 | 0.2386 | 0.2381 |
| OCL-LMN | Time | 4 | 0.0893 | 0.0223 | 11.5500 | <.0001 | <.0001 | <.0001 |
| OCL-LMN | Group*Time | 4 | 0.0115 | 0.0029 | 1.4800 | 0.2052 | 0.2181 | 0.2176 |
| ONB-LMN | Time | 4 | 0.2325 | 0.0581 | 25.3700 | <.0001 | <.0001 | <.0001 |
| ONB-LMN | Group*Time | 4 | 0.0204 | 0.0051 | 2.2200 | 0.0647 | 0.0768 | 0.0757 |
| TWOB-LMN | Time | 1 | 0.1410 | 0.1410 | 67.2700 | <.0001 |  |  |
| TWOB-LMN | Group*Time | 1 | 0.0004 | 0.0004 | 0.2000 | 0.6573 |  |  |
| GML-MPS | Time | 1 | 0.3201 | 0.3201 | 44.6500 | <.0001 |  |  |
| GML-MPS | Group*Time | 1 | 0.0205 | 0.0205 | 2.8600 | 0.0915 |  |  |

LMN= Speed is represented by the mean of the log10 transformed reaction times for correct responses (lower score= better performance); MPS= Moves per second; DET= Detection, IDN= Identification, OCL= One Card Learning, ONB= 1-Back, TWOB= 2-Back, GML= Groton Maze Learning (higher score= better performance); DF= Degree of Freedom; Type III SS= Type III Sum of Square adjusted for group, time, and group*time; G-G= Greenhouse-Geisser; H-F-L= Hu;ynh-Feldt-Lecoutre; Group, Time, and Group*Time were included in the model for repeated measure of Performance Speed dependent variables.

**eFigure 1.** **CBSB Measures for Accuracy of Performance by Study Groups across Timepoints**

**See eFigure file.**

Estimated mean score (95% Confidence Interval (CI)) in CogState measures across 5 sessions: ACC= Accuracy is represented by the arcsine transformation of the square root of the proportion of correct responses (higher score= better performance), TER= Total number of errors; DET= Detection, IDN= Identification, OCL= One Card Learning, ONB= 1-Back, TWOB- 2-Back, GML= Groton Maze Learning; *p<0.01

**eTable 6. CogState Measures for Accuracy of Performance by Study Groups**

|  |  | ME/CFS (n=261) | | HC (n=165) | | Difference | |
| --- | --- | --- | --- | --- | --- | --- | --- |
| Variable | Time | Mean | 95% CI^a^ | Mean | 95% CI | ES d^b^ | p-value |
| DET-ACC | T0 | 1.50 | (1.492,1.516) | 1.49 | (1.474,1.508) | 0.131 | 0.1991 |
| DET-ACC | T1 | 1.49 | (1.477,1.502) | 1.46 | (1.447,1.480) | 0.257 | 0.0117 |
| DET-ACC | T2 | 1.52 | (1.504,1.528) | 1.51 | (1.497,1.528) | 0.036 | 0.7522 |
| DET-ACC | T3 | 1.51 | (1.503,1.526) | 1.49 | (1.478,1.512) | 0.217 | 0.0521 |
| DET-ACC | T4* | 1.52 | (1.511,1.534) | 1.49 | (1.478,1.512) | **0.304** | **0.0072** |
| IDN-ACC | T0 | 1.42 | (1.409,1.440) | 1.42 | (1.406,1.443) | 0.000 | 0.9976 |
| IDN-ACC | T1 | 1.42 | (1.409,1.440) | 1.42 | (1.406,1.443) | 0.003 | 0.9741 |
| IDN-ACC | T2 | 1.47 | (1.454,1.488) | 1.45 | (1.434,1.474) | 0.148 | 0.1910 |
| IDN-ACC | T3 | 1.47 | (1.454,1.485) | 1.46 | (1.442,1.482) | 0.068 | 0.5356 |
| IDN-ACC | T4 | 1.46 | (1.442,1.478) | 1.46 | (1.443,1.487) | 0.036 | 0.7494 |
| OCL-ACC | T0 | 1.00 | (0.983,1.011) | 0.99 | (0.976,1.012) | 0.030 | 0.7726 |
| OCL-ACC | T1 | 1.01 | (0.999,1.026) | 1.02 | (1.000,1.039) | 0.058 | 0.5735 |
| OCL-ACC | T2 | 1.02 | (1.003,1.034) | 1.02 | (1.000,1.040) | 0.016 | 0.8911 |
| OCL-ACC | T3 | 1.01 | (0.998,1.031) | 1.03 | (1.007,1.047) | 0.101 | 0.3716 |
| OCL-ACC | T4 | 1.04 | (1.019,1.052) | 1.04 | (1.016,1.060) | 0.022 | 0.8489 |
| ONB-ACC | T0 | 1.38 | (1.367,1.401) | 1.38 | (1.361,1.407) | 0.000 | 0.9976 |
| ONB-ACC | T1 | 1.40 | (1.381,1.412) | 1.39 | (1.374,1.416) | 0.013 | 0.9003 |
| ONB-ACC | T2 | 1.42 | (1.401,1.438) | 1.42 | (1.403,1.447) | 0.040 | 0.7260 |
| ONB-ACC | T3 | 1.43 | (1.412,1.447) | 1.42 | (1.402,1.445) | 0.045 | 0.6932 |
| ONB-ACC | T4 | 1.42 | (1.398,1.434) | 1.42 | (1.397,1.443) | 0.034 | 0.7664 |
| TWOB-ACC | T0 | 1.23 | (1.211,1.255) | 1.24 | (1.211,1.264) | 0.029 | 0.7788 |
| TWOB-ACC | T1 | 1.24 | (1.222,1.265) | 1.25 | (1.223,1.282) | 0.048 | 0.6430 |
| GML-TER | T0 | 54.84 | (52.559,57.128) | 53.69 | (50.837,56.537) | 0.063 | 0.5332 |
| GML-TER | T1 | 51.69 | (49.466,53.913) | 51.49 | (48.629,54.359) | 0.011 | 0.9148 |

^a^CI= Confidence Interval; ^b^ES= Effect Size, Cohen’s d = 0.2 be considered a ‘small’ effect size, 0.5 represents a ‘moderate’ effect size and 0.8 a ‘large’ effect size; ACC= Accuracy is represented by the arcsine transformation of the square root of the proportion of correct responses (higher score= better performance); TER= Total number of errors; DET= Detection, IDN= Identification, OCL= One Card Learning, ONB= 1-Back, TWOB= 2-Back, GML= Groton Maze Learning

**eReferences**

Aitken, R. (1969). Measurement of feelings using visual analogue scales. Proc. R. Soc. Med. 62, 989–993.

Euroqol Q25 (2014). Europe quality of life, EQ-5D. Available online at: <https://euroqol.org/>

Rose, M., Bjorner, J., Becker, J., Fries, J., and Ware, J. (2008). Evaluation of a preliminary physical function item bank supported the expected advantages of the patient-reported outcomes measurement information system (PROMIS). J. Clin.Epidemiol. 61, 17–33. doi: 10.1016/j.jclinepi.2006.06.025

Devereaux Melillo, K., Williamson, E., Futrell, M., and Chamberlain, C. A. (1997). self-assessment tool to measure older adults’ perceptions regarding physical fitness and exercise activity. J. Adv. Nurs. 25, 1220–1226. doi: 10.1046/j.1365-2648.1997. 19970251220.x

British Columbia Ministry of Health (1978). Physical activity readiness questionnaire (PAR-Q) validation report. Victoria BC: British Columbia Ministry of Health.

Thomas, S., Reading, J., and Shephard, R. (1992). Revision of the physical activity readiness questionnaire (PAR-Q). Can. J. Sport Sci. 17, 338–345.

Booth, M. (2000). Assessment of physical activity: An international perspective. Res. Q. Exerc. Sport 71, 114–120. doi: 10.1080/02701367.2000.11082794

Craig, C., Marshall, A., Sjöström, M., Bauman, A., Booth, M., Ainsworth, B., et al. (2003). International physical activity questionnaire: 12-country reliability and validity. Med. Sci. Sports Exerc. 35, 1381–1395. doi: 10.1249/01.MSS.0000078924. 61453.FB

Ware, J., and Sherbourne, C. (1992). The MOS 36-item short-form health survey (SF-36). I. Conceptual framework and item selection. Med. Care 30, 473–483.

Ware, J. E. (2000). SF-36 health survey update. Spine (Phila Pa 1976) 25, 3130–3139. doi: 10.1097/00007632-200012150-00008

Smets, E., Garssen, B., Bonke, B., and De Haes, J. (1995). The Multidimensional Fatigue Inventory (MFI) psychometric qualities of an instrument to assess fatigue. J. Psychosom. Res. 39, 315–325. doi: 10.1016/0022-3999(94)00125-o

Wagner, D., Nisenbaum, R., Heim, C., Jones, J., Unger, E., and Reeves, W. (2005). Psychometric properties of the CDC Symptom Inventory for assessment of chronic fatigue syndrome. Popul. Health Metr. 3:8. doi: 10.1186/1478-7954-3-8

Cella, D., Yount, S., Rothrock, N., Gershon, R., Cook, K., Reeve, B., et al. (2007). The patient-reported outcomes measurement information system (PROMIS): Progress of an NIH roadmap cooperative group during its first two years. Med. Care 45, S3–S11. doi: 10.1097/01.mlr.0000258615.42478.55

Holdnack, J. A., Schoenberg, M. R., Lange, R. T., and Iverson, G. L. (2013). “Predicting premorbid ability for WAIS-IV, WMS-IV, and WASI-II,” in WAIS-IV, WMS-IV, and ACS, Academic Press, 217–278.
